# Predictors of Coronary Artery Disease in Heart Failure with Reduced Ejection Fraction at the Aga Khan University Hospital in Nairobi

**DOI:** 10.5334/gh.1271

**Published:** 2023-10-19

**Authors:** Redemptar Kimeu, Mohamed Jeilan, Mzee Ngunga

**Affiliations:** 1 Department of Cardiology, Aga Khan University Hospital, Nairobi, Kenya

**Keywords:** coronary artery disease, predictors, HFrEF

## Abstract

There appears to be an epidemiological transition in the etiology of heart failure in sub-Saharan Africa (SSA) in parallel with a steady increase in risk factors for coronary artery disease (CAD). SSA has limited access to heart failure and CAD diagnostics, limiting the number of patients who receive optimal care. Our objectives were to study the predictors of coronary artery disease among patients with heart failure with reduced ejection fraction (HFrEF) and develop a model to assist clinicians in determining the likelihood of CAD before cardiac catheterization.

**Methodology:**

This was a retrospective study at the Aga Khan University Hospital, Nairobi, which is equipped with diagnostic capabilities for heart failure and coronary artery assessment. We evaluated patients with HFrEF based on echocardiographic data over a 12-year period. Patients with coronary anatomical evaluation data were included. A multivariable model of CAD was generated using stepwise logistic regression.

**Results:**

Of the 1329 patients screened, 514 met the inclusion criteria. The mean age was 61.0 ± 12.8 years. There were 381 male cases (75.2%), and the predominant race was African, numbering 386 (75.2%). Most patients, 97%, were evaluated through conventional coronary angiography. Further, 310 (60.3%) cases had significant CAD. The prevalence of CAD in HFrEF was 52.3% in Africans, 85% in Asians, and 79% in Caucasians. In the multivariable logistic regression, the odds of having significant CAD was higher among participants with diabetes mellitus (aOR: 1.86; 95%CI: 1.15–3.03), Q waves (aOR: 2.12; 95%CI: 1.12–4.10), significant ST segment deviation (aOR: 4.14; 95%CI: 2.23–8.03), and regional wall motion abnormalities on echocardiogram (aOR: 6.53; 95%CI: 3.94–11.06).

**Conclusion:**

In this population, CAD was a major etiology in HFrEF among the African population. The most powerful predictors of CAD were type 2 diabetes, the presence of pathological Q waves, or ST segment shift on a 12-lead electrocardiogram, and regional wall motion abnormality on 2D echocardiogram.

**Highlights:**

1. There is an epidemiological transition in the cause of heart failure in sub-Saharan Africa (SSA) in keeping with the steady increase in cardiovascular risk factors for coronary artery disease (CAD).
2. The prevalence of CAD in African patients with heart failure with reduced ejection fraction (HFrEF) was 52.3%.
3. In the multivariable logistic regression, diabetic mellitus, pathological Q waves, significant ST segment deviation, and regional wall motion abnormalities were significantly associated with CAD.

## Background

Cardiovascular diseases (CVD) are among the leading causes of non-communicable diseases across Sub-Saharan Africa (SSA) accounting for 7% to 10% of all medical admissions to hospital, with heart failure contributing up to 3% to 7% [1,2]. Heart failure in SSA has a considerable socioeconomic impact due to its high prevalence, the high cost of hospitalizations (approaching 1,000 USD per patient in low-income countries), and a high mortality rate of up to 34% annually, as well as the relatively younger age of onset with the consequent involvement of economically active individuals [3,4]. The absolute number of CVD deaths has increased by more than 50% in the past three decades in SSA [5].

Heart failure with reduced ejection fraction (HFrEF) has ischemic and non-ischemic etiologies, and the extent of CAD often determines the pace of development and progression of ischemic cardiomyopathy [6]. Clinical guidelines recommend cardiac catheterization for newly diagnosed heart failure patients [7,8]; however, this is beyond reach for many patients in SSA.

The demographics surrounding HFrEF in SSA are known to be different from those in other parts of the world. However, there appears to be an epidemiological transition in the etiology of heart failure in SSA in parallel with a steady increase in risk factors for CAD. Most studies in SSA are based on electrocardiography and echocardiography criteria of ischemic heart disease (IHD). Data from 12 clinical studies performed before 2005 in eight SSA countries have shown that up to 75% of cases of heart failure were non-ischemic in origin [9,10]. In a multicenter study of the etiologies of acute heart failure in nine SSA countries, Damasceno et al. found that IHD was not a common cause, accounting for 7.7%. However, they excluded patients with acute ST elevation myocardial infarction [11]. Yuyun et al. noted that IHD was the most frequent cause of CVD death in SSA: 5% of all deaths and 40% of cardiovascular disease deaths [12]. In a TaHeF study looking at the etiology, clinical characteristics, and prognosis of adults with heart failure observed in a tertiary hospital in Tanzania, Makubi et al. found the prevalence of IHD at 9% [13].

However, with lifestyle changes that emulate the Western world, SSA may face a fundamental shift in the contributors to HFrEF away from hypertension or valvular heart disease and instead toward CAD [14]. In addition, SSA has limited access to both heart failure and CAD diagnostics, and therapeutic interventions, limiting the number of patients who receive optimal care. The resources for the treatment of heart failure in SSA are limited, thus the need to identify the right patient to refer for catheterization. There are multiple, validated risk scores to estimate the odds of coronary disease, though these were done in populations with different demographic characteristics and a higher prevalence of CAD as compared to the SSA population.

The objective of this study was to look at the predictors of coronary artery disease among patients with heart HFrEF in a single center in SSA and develop a model to assist clinicians in determining the likelihood of CAD before cardiac catheterization.

## Methodology

**Figure 1:**
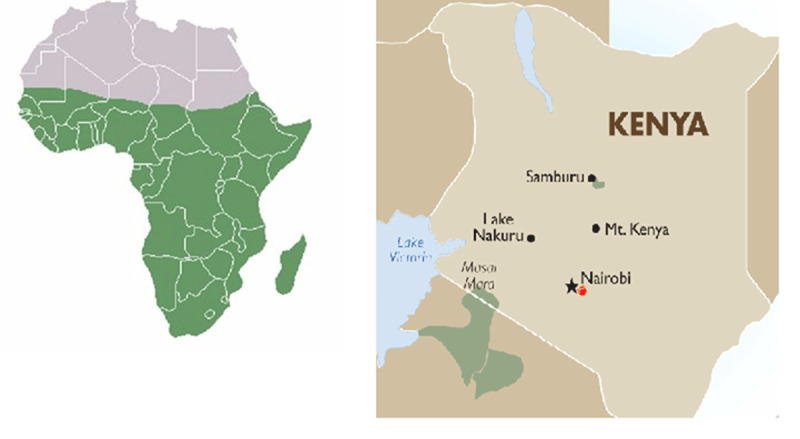
Map of Sub-Sahara Africa and Kenya. Map of Sub-Sahara Africa and Kenya. The Aga Khan University Hospital is a private 300 bed referral Centre located in Nairobi, Kenya’s capital city, receiving patients from the Aga khan outreach clinics in East Africa and other hospital both private and public in the East African community, a conglomerate of 10 countries. It is equipped with various modalities of cardiac imaging; an echocardiography lab, CT coronary angiography, Cardiac MRI and ischemia testing. It has two operational catheterization laboratories and is accredited as a primary PCI Centre. It has a dedicated coronary care unit as well as a 5-day outpatient cardiac clinic for ambulatory patients. It serves a multiracial community.

This was a retrospective study carried out at the Aga Khan University Hospital in Nairobi, a referral center that receives patients from the Aga Khan outreach clinics in East Africa and other hospitals, both private and public in the East African community, a conglomerate of 10 countries (Figure 1). It is adequately equipped with various modalities of cardiac imaging, as well as ischemia testing, such as an echocardiography lab, both an exercise and a dobutamine stress echocardiogram, nuclear imaging, coronary CT angiography, cardiac MRI and cardiac stress MRI. It has two operational catheterization laboratories and is accredited as a primary PCI center. It has a dedicated coronary care unit, as well as a five-day outpatient cardiac clinic for ambulatory patients. It has a full-fledged cardiovascular unit with capabilities of performing coronary artery bypass surgery. It serves a multiracial community, privately insured patients, and self-paying patients.

### Study population

All patients above 18 years of age with diagnosis of HFrEF based on echocardiography from January 2010 to March 2022 were included. We searched through hospital records, as well as through the catheterization laboratory database for evidence of either a conventional coronary angiogram or a computed tomography coronary angiogram within 12 months of HFrEF diagnosis until we achieved our target study population.

CAD was diagnosed in the presence of any significant epicardial coronary vessels stenosis, any history of MI, or coronary revascularization (either percutaneous trans-luminal coronary angioplasty or coronary artery bypass grafting) [15]. Lesions in an epicardial coronary artery were considered significant if ≥70% stenosis of the examined vessel or ≥50% of left main coronary artery (LMCA).

Demographic, clinical characteristics, and electrocardiographic data for patients were obtained at the time of heart failure diagnosis. The variables studied were;

- Gender
- Self-reported ethnicity
- Tobacco smoking history
- High-density lipoprotein cholesterol (HDL-C) level < 40 mg/dl (1.04 mmol/L)
- Systemic hypertension (blood pressure > 140/90 or use of antihypertensive medication
- Diabetes mellitus (DM), Use of insulin and duration of insulin use
- Family history of premature CAD (defined as a history of myocardial infarction in a first-degree relative)
- Estimated glomerular filtration rate,
- Hemoglobin
- Body mass index
- Peripheral artery disease
- Urban dwelling, defined as living in a place of any size within a densely settled area of at least 50,000 people and at least 1,000 people per square
- Sedentary lifestyle
- Alcohol intake.

A sedentary lifestyle was defined as those patients whose lifestyle did not meet the WHO recommendations [16] for physical activity and was dependent on physician assessment of physical activity. Regular alcohol use was defined as consumption three or more times a week.

Electrocardiographic variables included the presence or absence of bundle branch block (left or right), and the presence or absence of significant Q waves defined as any Q wave in leads V2–V3 ≥ 0.02 s, or QS complex in leads V2 and V3, Q wave ≥ 0.03 s and >0.1 mV deep, or QS complex in leads I, II, aVL, aVF, or V4–V6 in any two leads of a contiguous lead grouping (I, aVL,V6; V4–V6; II, III, and aVF) R-wave ≥ 0.04 s in V1–V2 and R/S ≥ 1 with a concordant positive T-wave in the absence of a conduction defect [17].

Significant ST changes were defined as ST-segment elevation ≥ 2.5 mm in men < 40 years, ≥2 mm in men ≥ 40 years, or ≥ 1.5 mm in women in leads V_2_–V_3_ and/or ≥ 1 mm in the other leads (in the absence of left ventricular (LV) hypertrophy or left bundle branch block LBBB)), and horizontal or down slopping ST depression > or = to 0.5 mm in two contiguous leads [18].

### Sample size determination

A minimum sample size of 514 patients was required to determine the predictors of CAD in HFrEF with 80% power using sample size formula for logistic regression. Other parameters were assumed as defined above, and a 5% nonresponse rate was considered among files with missing data due to incomplete records [19].

### Ethical considerations

This study was approved by the Aga Khan University Hospital ethical committee and relevant administrative bodies and conforms to the principles outlined in the Declaration of Helsinki. Patient identifiers were not included during data collection.

### Data analysis

Baseline characteristics were presented for the entire population, stratified according to the presence or absence of CAD. Continuous variables were described using the median (interquartile range (IQR)), while categorical variables were summarized using frequencies and percentages. The Wilcoxon rank sum test and Pearson’s chi-square or Fisher’s exact test were used to compare continuous and categorical variables, respectively. A multivariable logistic regression was performed to identify factors associated with the binary outcome of significant CAD. Any predictor with a Wald test *p*-value < 0.25 in the bivariate analysis or had known clinical significance was included in the multivariable logistic regression. The estimated values from the regression analysis were utilized to construct a nomogram.

A nomogram is a statistical tool designed to predict the likelihood of the outcome of interest and expresses the risk factors for diseases based on the characteristics of patients. The location and length of each nomogram line illustrates its relative importance with respect to the risk of significant CAD. The sum of the predictors yields the ‘total score’, which can be scaled to the final output probability of significant CAD.

All the statistical analyses were conducted using the statistical package for social science (SPSS) software, version 23 (IBM Corp. Armonk, NY), with a significance level set at *p*-value < 0.05.

## Results

### Baseline characteristics of the patients

A total of 514 participants with LV dysfunction were identified and included in the study. Of the participants identified, 310 (60.3%) had significant CAD. Overall, the median age was 62.0 (IQR: 52.0–71.0) years, males were 381 (74.1%), and 386 (75.1%) were of African origin. The overall median BMI was 27.1 (IQR: 24.5–30.4) kg/m^2^. Sixty-nine (15.7%) of the participants were consumers of alcohol, and a majority were in NYHA II at the time of the first encounter (Table 1).

**Table 1:** Baseline characteristics stratified by significant CAD status, N = 514.


VARIABLE	OVERALL, N = 514^1^	SIGNIFICANT CAD STATUS		P-VALUE^2^

		WITHOUT SIGNIFICANT CAD, N = 204^1^	WITH SIGNIFICANT CAD, N = 310^1^	

**Age in years**	62.0(52.0, 71.0)	57.0(49.0, 67.0)	64.0(56.0, 72.0)	<0.001

**Gender**				<0.001

Males	381/514(74.1%)	134/204(65.7%)	247/310(79.7%)	

Females	133/514(25.9%)	70/204(34.3%)	63/310(20.3%)	

**Race**				<0.001

Caucasian	20/514(3.9%)	4/204(2.0%)	16/310(5.2%)	

African	386/514(75.1%)	184/204(90.2%)	202/310(65.2%)	

Asian	108/514(21.0%)	16/204(7.8%)	92/310(29.7%)	

**height (cm)**	169.0(161.0, 175.5)	168.0(161.0, 176.0)	170.0(162.0, 175.0)	0.46

**weight (Kg)**	78.0(69.0, 89.0)	79.0(70.0, 89.2)	78.0(66.6, 89.0)	0.34

**BMI Kg/m** ^2^	27.1(24.5, 30.4)	27.6(25.2, 31.0)	26.7(24.1, 30.3)	0.025

**Dwelling**				<0.001

Urban	216/507(42.6%)	64/202(31.7%)	152/305(49.8%)	

Rural	36/507(7.1%)	20/202(9.9%)	16/305(5.2%)	

Unknown	255/507(50.3%)	118/202(58.4%)	137/305(44.9%)	

**Lifestyle**				0.040

Sedentary	184/473(38.9%)	75/188(39.9%)	109/285(38.2%)	

Non-sedentary	49/473(10.4%)	27/188(14.4%)	22/285(7.7%)	

Unknown	240/473(50.7%)	86/188(45.7%)	154/285(54.0%)	

**Alcohol intake**	69/439(15.7%)	31/183(16.9%)	38/256(14.8%)	0.55

**NYHA Classification at first encounter**				0.035

NYHA I	36/500(7.2%)	17/200(8.5%)	19/300(6.3%)	

NYHA II	294/500(58.8%)	104/200(52.0%)	190/300(63.3%)	

NYHA III	104/500(20.8%)	53/200(26.5%)	51/300(17.0%)	

NYHA IV	66/500(13.2%)	26/200(13.0%)	40/300(13.3%)	


^1^ Median (IQR); n/N(%). ^2^ Wilcoxon rank sum test; Pearson’s Chi-squared test.

Baseline characteristics stratified by the absence or presence of significant CAD are shown in Table 1. There were 498 (96.8%) cases with a conventional coronary angiogram, and 3.2% had a CT coronary angiogram. There was a significant difference between those with CAD and those without in terms of age, gender, race, BMI, place of dwelling, and NYHA classifications. Participants with significant CAD were significantly older, male, dwelling in urban areas, and had a functional class of NYHA II compared to those without significant CAD. Participants without significant CAD were more likely of African origin, with a higher BMI and a sedentary lifestyle. The prevalence of CAD in HFrEF was 52.3% in Africans, 85% in Asians and 79% in Caucasians. The majority of cases, 257 (86%), had involvement of left anterior descending artery disease, while 22% had triple vessel disease. Only 2.5% had involvement of the left main.

### Risk factors for CAD

Most of the participants reported systemic hypertension as a risk factor at 65.4% (*n* = 336); this was followed by BMI > 25 kg/m^2^ at 54.7% (*n* = 281). The least common risk factors reported were a family history of CAD (2.5%; *n* = 13) and triglycerides > 5.17 mmol/L—reported by only two participants. The proportion of participants reporting diabetes mellitus, age > 55 years for women, age > 45 years for men, and BMI > 25 kg/m^2^ as a risk factor were significantly higher in participants with significant CAD than their counterparts without significant CAD (Table 2). The proportion of current tobacco smokers, users of insulin, and those with a history of NSTEMI and angina were significantly higher in participants with significant CAD compared to those without significant CAD (Table 2).

**Table 2:** Traditional risk factors stratified by significant CAD status, N = 514**.


VARIABLE	OVERALL, N = 514^1^	SIGNIFICANT CAD STATUS		P-VALUE^2^

		WITHOUT SIGNIFICANT CAD, N = 204^1^	WITH SIGNIFICANT CAD, N = 310^1^	

**Systemic hypertension**	336/514(65.4%)	134/204(65.7%)	202/310(65.2%)	0.90

**Diabetes mellitus**	242/514(47.1%)	80/204(39.2%)	162/310(52.3%)	0.004

**Hypercholesterolemia**	65/514(12.6%)	22/204(10.8%)	43/310(13.9%)	0.30

**Tobacco Smoking history**	71/514(13.8%)	26/204(12.7%)	45/310(14.5%)	0.57

**Family history of CAD first degree relative < 65 years**	13/514(2.5%)	3/204(1.5%)	10/310(3.2%)	0.21

**Age > 55 years women**	100/514(19.5%)	49/204(24.0%)	51/310(16.5%)	0.034

**Age > 45 years men**	321/514(62.5%)	106/204(52.0%)	215/310(69.4%)	<0.001

**Low HDL < 1.0 mmol/l**	141/514(27.4%)	48/204(23.5%)	93/310(30.0%)	0.11

**TGS > 5.17 mmol/l**	2/514(0.4%)	0/204(0.0%)	2/310(0.6%)	0.52

**BMI > 25 kg/m^2^**	281/514(54.7%)	128/204(62.7%)	153/310(49.4%)	0.003


^1^ n/N(%). ^2^ Pearson’s Chi-squared test; Fisher’s exact test.

### Electrocardiographic characteristics

Of the participants included in the study, 122 (23.7%) had Q waves on electrocardiogram, 161 (31.3%) had significant ST segment deviation, 31 (6.0%) had atrial fibrillation or flutter, and 69 (13.4%) had LBBB. The proportion with Q waves was 32.9% (*n* = 120) among those with significant CAD, compared to 9.8% (*n* = 20) in those without CAD (*p*-value < 0.001). The proportion with significant ST segment deviation among patients with significant CAD and among those without significant CAD was 46.1% (*n* = 143) and 8.8% (*n* = 18), respectively. The proportion of participants with atrial fibrillation or flutter among the patients without significant CAD was significantly higher than those with significant CAD (9.3% vs 3.9%; *p*-value = 0.011).

### Echocardiographic characteristics

Overall the mean LVEF was 28.47% ± 8.77. The mean left ventricular end diastolic dimension (LVEDD) and left ventricular end systolic dimension (LVEDD) were 53.72 mm ± 9.07 and 45.66 mm ± 10.25 mm, respectively. The LVEDD and LVESD were significantly higher in patients without significant CAD; 55.66 mm (±9.55) and 47.73 mm (±11.16), respectively, compared to those with significant CAD, *p*-values < 0.001. A finding of regional wall motion abnormalities, was significantly associated with significant CAD: 208 cases (67.1%) vs 36 cases (17.6%) in non-significant CAD, *p*-value < 0.001. Global hypokinesia, with 181 (42.6%) cases overall, was significantly associated with non-significant CAD: 120 (83.3%) cases, *p*-value < 0.001. Moderate to severe mitral regurgitation was found in 122 (23.7%) of the patients. There was a trend toward more cases of severe mitral regurgitation having non-significant CAD, at 8.1% compared to 3.7% in those with significant CAD. Moderate to severe aortic regurgitation was present in 2.3% of the patients. Overall the mean pulmonary pressure was 42.49 mmHg ± 17.76 (Table 3).

**Table 3:** 2D Echocardiographic and MRI characteristics by CAD, N = 514**.


VARIABLE	OVERALL, N = 514^1^	SIGNIFICANT CAD STATUS		P-VALUE^2^

		WITHOUT SIGNIFICANT CAD, N = 204^1^	WITH SIGNIFICANT CAD, N = 310^1^	

**LVEDD**	54.0(47.0, 59.0)	56.0(49.0, 62.0)	52.0(47.0, 58.0)	<0.001

**LVESD**	46.0(38.0, 52.0)	48.0(40.0, 56.0)	43.0(37.0, 50.0)	<0.001

**Modified biplane simpson’s rule LVEF**	30.0(20.0, 35.0)	25.0(20.0, 35.0)	33.0(25.0, 35.0)	<0.001

**LV mass index**	123.9(98.3, 153.1)	129.2(102.8, 164.4)	119.4(97.4, 143.2)	0.042

**Regional Wall motion**	244/514(47.5%)	36/204(17.6%)	208/310(67.1%)	<0.001

**MRI**	46/494(9.3%)	23/194(11.9%)	23/300(7.7%)	0.12

**MRI-LVEF**	35.0(26.0, 40.0)	34.0(23.8, 39.0)	35.0(26.0, 45.5)	0.55

**MRI-RVEF**	47.0(34.5, 58.5)	44.0(34.0, 48.0)	57.0(38.0, 66.0)	0.025

**MRI-LGE**	35/45(77.8%)	13/22(59.1%)	22/23(95.7%)	0.004

**MRI-Ischaemic Cardiomyopathy**	25/45(55.6%)	4/22(18.2%)	21/23(91.3%)	<0.001


^1^ Median (IQR); n/N(%). ^2^ Wilcoxon rank sum test; Pearson’s Chi-squared test; Fisher’s exact test.

The mean left ventricular mass index (LV mass index) for males and females were 133 g/m^2^ (44.4) and 116 g/m^2^ (40.0), respectively. Females with an LV mass index > 95 g/m^2^ were 42 (38.9%) and males with an LV mass index > 115 g/m^2^ were 169 (43.8%) (Table 3).

### The predictors of CAD in HFrEF

Selected demographic, clinical, and echocardiographic variables were adjusted in the logistic regression model to identify factors associated with significant CAD. In the multivariable analysis, having DM, pathological Q waves on electrocardiogram, significant ST segment deviation, and regional wall motion abnormality were found to be risk factors associated with significant CAD, whereas having a BMI > 25 kg/m^2^ was inversely associated with significant CAD. The odds of having significant CAD were almost twice as high among participants with DM than those without DM (aOR: 1.86; 95%CI: 1.15–3.03). Participants with Q waves on electrocardiogram were 2.12 times more likely to have significant CAD compared to those without Q waves on electrocardiogram (aOR: 2.12; 95%CI: 1.12–4.10). The likelihood of significant CAD was 4.14 times higher among participants with ST segment deviation than in their counterparts without ST segment deviation (aOR: 4.14; 95%CI: 2.23–8.03), and having RWMA was associated with 6.53 times the odds of having significant CAD than without RWMA (aOR: 6.53; 95%CI: 3.94–11.06). Finally, having a BMI > 25 kg/m^2^ was associated with 52% lower risk of having a significant CAD. Though not significantly associated with significant CAD, age > 55 years for women and age > 45 years for men increased odds of having significant CAD (Table 4).

**Table 4:** Multivariable logistic regression model for patients with significant CAD.


		UNIVARIABLE		MULTIVARIABLE	
	
		OR (95%CI)	P-VALUE	OR (95%CI)	P-VALUE

Gender	Males	1			

	Females	0.49 (0.33–0.73)	<0.001	0.65 (0.29–1.49)	0.311

Race	Caucasian	1			

	African	0.27 (0.08–0.76)	0.023	0.45 (0.10–1.71)	0.267

	Asian	1.44 (0.38–4.55)	0.559	1.60 (0.32–6.83)	0.547

LVEF		1.06 (1.04–1.08)	<0.001	1.02 (0.99–1.05)	0.136

Current tobacco smoker	Yes	3.41 (1.48–9.23)	0.007	2.43 (0.86–7.70)	0.108

	No	1			

DM	Yes	1.70 (1.19–2.43)	0.004	1.86 (1.15–3.03)	0.011

	No	1			

Women: age > 55 years	Yes	0.62 (0.40–0.97)	0.035	1.36 (0.59–3.19)	0.475

	No	1			

Men: age > 45 years	Yes	2.09 (1.45–3.02)	<0.001	1.64 (0.81–3.35)	0.168

	No	1			

BMI > 25 (Kg/m^2^)	Yes	0.58 (0.40–0.83)	0.003	0.48 (0.29–0.77)	0.003

	No	1			

Q waves	Yes	4.51 (2.74–7.77)	<0.001	2.12 (1.12–4.10)	0.022

	No	1			

ST segment	Yes	8.85 (5.32–15.52)	<0.001	4.14 (2.23–8.03)	<0.001

	No	1			

LBBB	Yes	0.34 (0.20–0.57)	<0.001	1.20 (0.52–1.90)	0.993

	No	1			

Regional Wall motion	Yes	9.52 (6.25–14.81)	<0.001	6.53 (3.94–11.06)	<0.001

	No	1			


**Figure 2:**
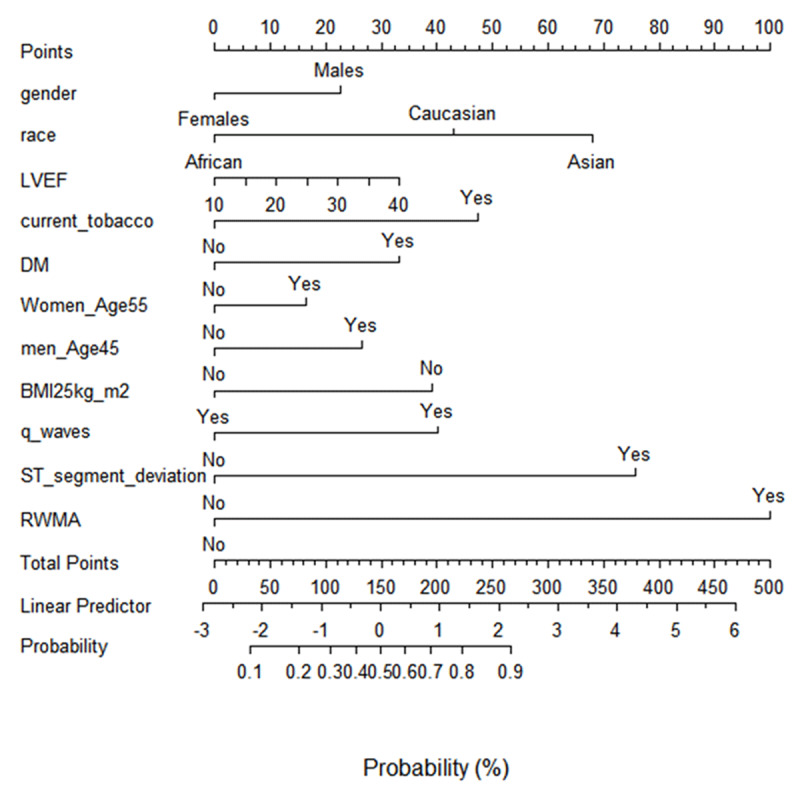
The nomogram for the logistic regression for predictors of coronary artery disease. LVEF-left ventricular ejection fraction, DM-diabetes Mellitus, LBBB- left bundle branch block, RWMA-regional wall motion abnormality. The location and length of each nomogram line illustrates its relative importance with respect to the risk of significant CAD. The sum of the predictors yields the “Total Score” which can be scaled to the final output probability of Significant CAD.

The nomogram for the logistic regression is shown in Figure 2. The predicted probability ranges from 0.1 to 0.9. The total points accumulated by the various covariates corresponds to the predicted probability for a patient. The point system works by ranking the effect estimates regardless of statistical significance, and it is influenced by the presence of other covariates. The higher the number of points, the more important the effect in explaining the outcome.

RWMA had the greatest contribution to the risk of significant CAD, and this was followed by significant ST segment deviations and race. Current tobacco smoking, DM, and pathological Q waves on electrocardiogram had modest contribution to the risk of significant CAD. whereas gender had the least contribution.

### Cardiac MRI findings

Forty-six cases underwent a cardiac MRI. Thirty-five cases (76%) had late gadolinium enhancement. Ischemic cardiomyopathy was diagnosed in 25 cases, of which 22 cases (88%) had coronary atherosclerosis on conventional coronary angiography. Non-ischemic cardiomyopathy was diagnosed in 20 cases, of which 70% had idiopathic cardiomyopathy, 25% had myocarditis, and 4% had amyloidosis (Table 3).

### Associated comorbidities

Of the patients, 32% (155 cases) had anemia, and there was no significant difference between patients with significant or non-significant CAD. The mean transferrin saturation was 19.84% ± 14.10. Overall, 322 (66.1%) cases had an estimated glomerular filtration rate greater than 60 ml/min/1.73 m^2^. There was no significant difference between patients with significant or non-significant CAD. The mean NT pro BNP for the study population was 8,824.31 pg/ml.

### Medications at discharge

At discharge, 442 (86%) patients were on B-blockers, 358 (69.6%) were on renin angiotensin aldosterone system blockers, 188 (36.5%) were on ARNI, and 270 (52.5%) on mineralocorticoid receptor blockers. Furthermore, 310 (99.6%) of patients with significant CAD were on a statin, 288 (92.6%) were on junior aspirin, and 230 (73.9) were on a P2Y12 inhibitor.

## Discussion

This study found that among HFrEF patients at the Aga Khan University Hospital in Nairobi with coronary angiographic studies as part of their work up, the prevalence of ischemic cardiomyopathy overall was 60.3%, and the prevalence in black Africans was 52.3%. In the multivariable logistic regression model, diabetic mellitus, pathological Q waves, a significant shift in the ST segment, and echocardiographic finding of regional wall motion abnormality were the predictors of CAD in patients with HFrEF. Patients without significant CAD had larger LVEDD and LVESD, which could be attributed to the duration of the LV remodeling process in non-ischemic cardiomyopathy prior to clinically significant symptoms and diagnosis.

Despite recent advances in care, IHD is a major contributor to the global disease burden. By 2017, the age-standardized death rate in SSA attributable to IHD was 50–70 per 100,000 [20]. In Kenya, the total CVD mortality rate was 13.8% in 2019 [21]. IHD is a major underlying pathologic process of heart failure, increasing the risk of heart failure eightfold, with a population-attributable risk of 65% in men and 48% in women [22].

We screened 1329 patients with echocardiographic evidence of HFrEF and included only patients who had undergone evaluation for coronary artery anatomy. The high prevalence of 52.3% in the African population might have been influenced by the selection bias of the patients due to the high cost of imaging, as well as cardiologists imaging patients who have a high pretest probability for CAD. The data seem to indicate a shift from previously published data from SSA, which have shown prevalence rates of about 10%. The published etiologies of heart failure in SSA are idiopathic cardiomyopathies, systemic hypertension, and valvular heart disease. However, data from previous studies was based on multiple echocardiographic studies in SSA performed before 2005, which showed that up to 75% of heart failure cases were non-ischemic in origin [10,11]. The Sub-Saharan Africa Survey of Heart Failure (THESUS-HF) study, employing noninvasive methods for establishing CAD, noted a rise in IHD from 2% to 8% as compared to African data before 2015 in the etiology of heart failure [11,23]. The Heart of Soweto was a prospective study, which showed a prevalence of 10% for ischemic cardiomyopathy. However, diagnosis was based on an initial clinical suspicion of CAD based on ECG (e.g., pathological Q waves) and echocardiography findings of regional wall motion abnormalities, as well as a combination of stress testing, cardiac nuclear imaging, and cardiac catheterization [24]. The population under study was younger with a mean age of 57 years ± 14, as compared to our patients, who had a mean age of 63 years ± 12, and only 67% underwent coronary angiographic studies.

The high prevalence noted in this study may be attributable to the different socioeconomic status of our study population as compared to studies done previously, as well as an increasing prevalence of major cardiovascular risk factors over the years [2,25]. From our study, the prevalence of ischemia among patients with HFrEF is similar to other parts of the world, which ranges from 39%–65% [26,27,28].

The predictors of significant CAD in patients with HFrEF are useful to guide physicians and systems of care when making decisions for expensive coronary artery angiographic studies. The most powerful variables (type 2 diabetes mellitus, pathological Q waves, shift in the ST segment, and regional wall motion abnormality on 2D echocardiography) are variables that can be predicted by clinical, electrocardiographic, or transthoracic echo assessment with a good sensitivity and specificity. These variables can be derived in a resource-limited health care system and could guide clinicians to select patients who would require further diagnostic cardiac catheterization. These CAD predictors are similar to previous studies done. Whellam et al., looking at the Duke University’s database, found that the predictors of CAD in heart failure were history of MI, age, diabetes mellitus, Q wave on electrocardiogram, male sex, regional wall motions abnormalities, race, history of angina, history of peripheral vascular disease, hyperlipidemia, and carotid bruits; this significantly predicted the presence of significant CAD in patients with LV dysfunction [29]. Previous studies done in the 1980s found echocardiography to be of limited value in distinguishing the underlying cause of LV dysfunction, however, these studies were limited by a small number of cases, and there has since been great advancement in echocardiographic techniques [30,31]. This study, however, had fewer predictors for CAD, a finding attributable to the challenges faced with incomplete data sets in retrospective studies.

The nomogram developed predicted the probability for CAD from 0.1 to 0.9. This simple tool can guide the appropriate guidelines for directed optimal medical therapy for HFrEF depending on predicted etiology. It further identifies patients who may benefit from coronary imaging and subsequent revascularization modalities, whether with percutaneous coronary intervention or coronary artery bypass grafting surgery. A prospective arm of this study is warranted to test the robustness of this predictive tool.

### Study limitations

This study had several limitations. The study cohort was identified at a single center, which might vary greatly with other centers in SSA in terms of the socioeconomic factors and health seeking behaviors of patients. The Aga Khan University Hospital Nairobi is one of very few primary PCI capable centers in the region with protocols adopted from the European Society of Cardiology (ESC) 2020 ACS in Patients Presenting without Persistent ST-Segment Elevation (Management of) and European Society of Cardiology (ESC) 2017 Management of Acute Myocardial Infarction in Patients Presenting with ST-Segment Elevation Guidelines. These protocols may differ from other centers in the region, and this may have created a selection bias.

The data was retrospectively collected, and challenges in data completeness and non-blinded interpretation of all cardiac imaging were faced. The study population was of patients who underwent coronary artery imaging, as this is the gold standard for determining CAD; other noninvasive modalities of diagnosis CAD were not included. As this was a retrospective study, the criteria used to refer patients with HFrEF for either invasive or noninvasive testing for ischemia was not clear. This poses a selection bias in our study population.

This study did not determine the number of patients with heart failure who did not undergo invasive coronary angiography or CT coronary angiography. Therefore, there is a possible selection bias, as some patients with HFrEF may not have had coronary angiography because of clinical or financial exclusion and thus, did not fulfill the inclusion criteria.

## Conclusion

This study demonstrates a higher prevalence of coronary artery disease among patients with heart failure than other studies in the region, demonstrating an epidemiological shift in the etiology of CAD. The predictors of CAD in our population were type 2 diabetes, pathological Q waves, ST segment shift on a 12-lead electrocardiogram, and regional wall motion abnormality on 2D echocardiogram. These predictors may be useful in guiding clinicians and patients within resource-limited settings in SSA in selecting which patients require coronary angiography in order to establish an underlying diagnosis of CAD.

## Additional File

The additional file for this article can be found as follows:

10.5334/gh.1271.s1 Dataset. This is the raw data the study demonstrating the variables studied for the 514 patients who met the inclusion criteria.
